# Interacting effects of unobserved heterogeneity and individual stochasticity in the life history of the southern fulmar

**DOI:** 10.1111/1365-2656.12752

**Published:** 2017-10-10

**Authors:** Stéphanie Jenouvrier, Lise M. Aubry, Christophe Barbraud, Henri Weimerskirch, Hal Caswell

**Affiliations:** ^1^ Biology Department Woods Hole Oceanographic Institution Woods Hole MA USA; ^2^ Centre d’Etudes Biologiques de Chizé, UMR 7372 CNRS Univ La Rochelle Villiers en Bois France; ^3^ Fish, Wildlife and Conservation Biology Department Colorado State University Fort Collins CO USA; ^4^ Institute for Biodiversity and Ecosystem Dynamics, University of Amsterdam Amsterdam The Netherlands

**Keywords:** frailty, individual quality, latent, life expectancy, lifetime reproductive success

## Abstract

1. Individuals are heterogeneous in many ways. Some of these differences are incorporated as individual states (e.g. age, size, breeding status) in population models. However, substantial amounts of heterogeneity may remain unaccounted for, due to unmeasurable genetic, maternal or environmental factors.
2. Such unobserved heterogeneity (UH) affects the behaviour of heterogeneous cohorts via intra‐cohort selection and contributes to inter‐individual variance in demographic outcomes such as longevity and lifetime reproduction. Variance is also produced by individual stochasticity, due to random events in the life cycle of wild organisms, yet no study thus far has attempted to decompose the variance in demographic outcomes into contributions from UH and individual stochasticity for an animal population in the wild.
3. We developed a stage‐classified matrix population model for the southern fulmar breeding on Ile des Pétrels, Antarctica. We applied multievent, multistate mark–recapture methods to estimate a finite mixture model accounting for UH in all vital rates and Markov chain methods to calculate demographic outcomes. Finally, we partitioned the variance in demographic outcomes into contributions from UH and individual stochasticity.
4. We identify three UH groups, differing substantially in longevity, lifetime reproductive output, age at first reproduction and in the proportion of the life spent in each reproductive state.
  –14% of individuals at fledging have a delayed but high probability of recruitment and extended reproductive life span.
  –67% of individuals are less likely to reach adulthood, recruit late and skip breeding often but have the highest adult survival rate.
  –19% of individuals recruit early and attempt to breed often. They are likely to raise their offspring successfully, but experience a relatively short life span.
  Unobserved heterogeneity only explains a small fraction of the variances in longevity (5.9%), age at first reproduction (3.7%) and lifetime reproduction (22%).
5. UH can affect the entire life cycle, including survival, development and reproductive rates, with consequences over the lifetime of individuals and impacts on cohort dynamics. The respective role of UH vs. individual stochasticity varies greatly among demographic outcomes. We discuss the implication of our finding for the gradient of life‐history strategies observed among species and argue that individual differences should be accounted for in demographic studies of wild populations.

## 1 INTRODUCTION

In any population, individuals differ in many of their life‐history characteristics. One task of demographers is to incorporate the most important of these differences into the *individual state* (i‐state) of a structured population model (Caswell, 2001; Metz & Diekmann, 1986). Individual states may be based on age, size, developmental state, reproductive condition or other life‐history characteristics. The resulting models have been widely used to address questions in conservation and wildlife management, epidemiology, ecotoxicology and evolutionary ecology. However, even after taking i‐state differences into account, differences may remain among individuals of the same age, size, state, etc. Such residual heterogeneity has been given many names: latent, unobserved individual heterogeneity (Cam, Link, Cooch, Monnat, & Danchin, 2002; Link, Cooch, & Cam, 2002), frailty in survival analysis (Vaupel & Yashin, 1985; Vaupel, Manton, & Stallard, 1979) or individual quality in studies of reproductive parameters (Wilson & Nussey, 2010). Herein, we adopt the general term *unobserved heterogeneity* (UH) in vital rates (i.e. survival and reproductive rates) to refer to unobserved differences among individuals, regardless of which vital rates they affect. Such differences may be fixed or may change dynamically over the life of an individual; here, we consider the case of fixed heterogeneity (e.g. Vaupel et al., 1979).

Variance among individuals in their demographic performance arises from observed heterogeneity (e.g. differences due to age, sizes, developmental stage), UH and individual stochasticity. Indeed, every life cycle contains probabilistic events such as living or dying, recruiting or not, breeding or failing, etc. Because of these random events, demographic outcomes can vary due of chance alone, a source of variance called *individual stochasticity* (Caswell, 2009, 2011, 2014; van Daalen & Caswell, 2015). Individual stochasticity can produce variance in demographic outcomes even when all individuals are identical and experience the same vital rates at every age or stage. Individual stochasticity has been quantified for longevity (Caswell, 2006, 2009, 2014), stage occupancy times (Caswell, 2006) and lifetime reproduction (Caswell, 2011; Steiner & Tuljapurkar, 2012; Steiner, Tuljapurkar, & Orzack, 2010; Tuljapurkar & Steiner, 2010; Tuljapurkar, Steiner, & Orzack, 2009; van Daalen & Caswell, 2015, 2017).

Thus, inter‐individual variance in demographic outcomes is not, by itself, evidence for heterogeneity. Rather, it is the combined effect of UH and individual stochasticity. To evaluate the relative contributions of heterogeneity and stochasticity requires a stochastic analysis of a demographic model that incorporates both. Such a model estimates the nature and degree of UH and includes it in the state space of a multistate matrix population model. The variance produced by this multistate model can then be decomposed into contributions from the two sources (Cam et al., 2013; Cam, Aubry, & Authier, 2016; Caswell, 2014; Hartemink, Missov, & Caswell, 2017; van Daalen & Caswell, 2015), which is the procedure we followed here.

A variety of genetic, maternal and environmental factors can lead to UH (Wilson & Nussey, 2010). When UH involves survival, it produces changes in the composition of a cohort as it ages (Vaupel et al., 1979). Frail individuals tend to die sooner, leaving the cohort progressively composed of more robust individuals. Population‐level patterns of age‐specific survival from such a cohort are distorted by within‐cohort selection (Vaupel & Yashin, 1985; Vaupel et al., 1979). Within‐cohort selection may also result in positive covariation among life‐history traits at the individual level (e.g. longevity and breeding probability, Cam et al. (2002); current and future reproductive success, Aubry, Koons, Monnat, and Cam (2009); age‐specific survival and reproductive success, Aubry, Cam, Koons, Monnat, and Pavard (2011)) because when frail individuals die, associated reproductive traits disappear from the population with them.

Many studies in human demography (Aalen, 1994; Hougaard, 1995; Vaupel et al., 1998; Yashin & Iachine, 1995) and in ecology (Aubry et al., 2011; Cam et al., 2002; Cam et al., 2013; Fox, Kendall, Fitzpatrick, & Woolfenden, 2006; Wintrebert, Zwinderman, Cam, Pradel, & van Houwelingen, 2005) have detected substantial UH in survival, with some individuals experiencing lower mortality (“robust” individuals) than others (“frail” individuals). Yet, UH in reproductive parameters has not received as much attention as frailty in demographic and life‐history studies (but see Bouwhuis, Sheldon, Verhulst, & Charmantier, 2009; Chambert, Rotella, Higgs, & Garrott, 2013; Chambert, Rotella, & Garrott, 2014; Rebke, Coulson, Becker, & Vaupel, 2010).

We analyse the relative contributions of heterogeneity and stochasticity to life‐history outcomes for an ice‐dependent Antarctic seabird, the southern fulmar (*Fulmarus glacialoides*). To do so, we first develop a structured life cycle model whereby the i‐states are based on reproductive status, and are parameterized in terms of stage‐specific survival, breeding probability and breeding success (Jenouvrier, Peron, & Weimerskirch, 2015). Second, we estimate UH in all of these vital rates using multievent finite mixture models that account for UH within a capture–mark–recapture framework (Hamel, Yoccoz, & Gaillard, 2017; Peron et al., 2010; Pradel, 2005), assuming UH is fixed over the life cycle of the southern fulmar. Third, we use Markov chain methods to calculate the inter‐individual variance in longevity, lifetime reproductive output (LRO), age at maturity and inter‐breeding intervals (Caswell, 2001, 2006, 2009) for each identified UH group. The identified UH groups define sets of life‐history characteristics that occur together within a life history; we refer to these as *life‐history complexes*. Finally, the demographic properties of the population are determined by the mixture distribution of identified life‐history complexes; we use our demographic model of a heterogeneous cohort to decompose the variances in longevity, LRO and age at first reproduction into contributions due to UH and individual stochasticity.

## 2 STUDY SPECIES: THE SOUTHERN FULMAR

The southern fulmar (*F. glacialoides*) breeds in the Southern Hemisphere along the mainland coast of Antarctica and on nearby islands, and migrates to sub‐Antarctic and subtropical waters during the non‐breeding season (Delord et al., 2016). It breeds during the austral summer from October to March; a single egg is laid per breeding season. Fulmars feed mainly on krill (e.g. *Euphausia superba*) and other crustaceans, as well as on small fish (*Pleuragramma antarctica*) and carrion (Ridoux & Offredo, 1989).

Our study population is located on Ile des Pétrels, (66^°^40^′^S, 140^°^01^′^E), Antarctica. Mark–recapture data have been collected on this population since 1962. We utilized data from 1964 to 2010 on known‐age individuals banded as fledglings (*n* = 1,165 individuals). For more details on the study population and banding protocol, see Jenouvrier, Barbraud, and Weimerskirch (2003).

## 3 DEMOGRAPHY AND HETEROGENEITY

### 3.1 The fulmar life cycle

**Figure 1:**
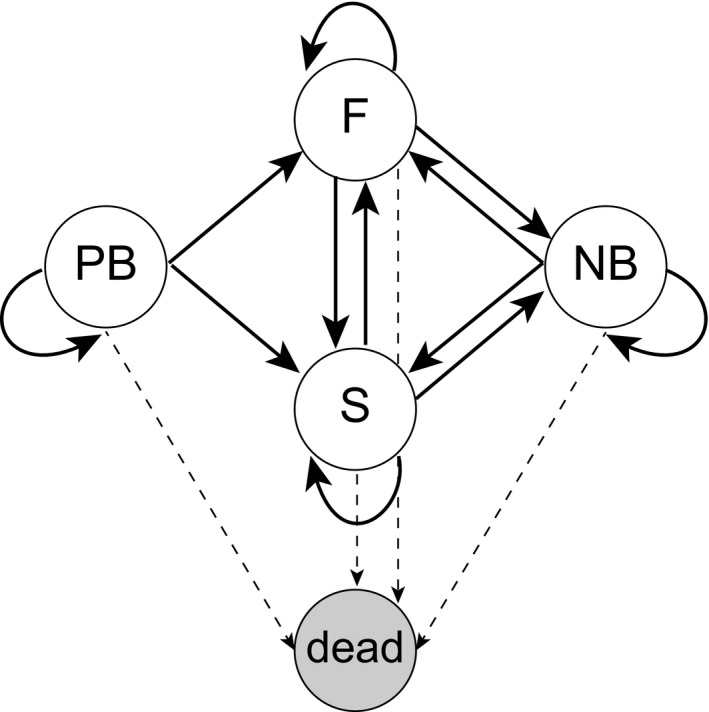
Life cycle graph for the southern fulmar. Projection interval is 1 year. Nodes correspond to states: PB = pre‐breeders; S = successful breeders; F = failed breeders; NB = non‐breeders. Solid arcs indicate transitions among surviving individuals, while dashed lines show transitions to the absorbing state of death

Our analysis is based on a life cycle that includes four stages (*s* = 4), based on breeding states defined at the end of the breeding season (Figure 1).

1. Pre‐breeders: individuals that have yet to breed; this stage includes fledged chicks produced during the current season.
2. Successful breeders: individuals that successfully raised a chick during the current season.
3. Failed breeders: individuals that either failed to hatch an egg or failed to raise a chick during the current season.
4. Non‐breeders: individuals that have bred at least once before, but did not breed in the current season.

The annual life cycle starts in March of year *t*, immediately after the fledging period. The vital rates associated with the life cycle transitions among states are:

1. Stage‐specific survival probability σ_*j*_: the probability of surviving and not permanently emigrating to a different colony from the end of the breeding season in one year to the end of the breeding season in the next year.
2. Stage‐specific breeding probability β_*j*_: the conditional probability of returning to the colony and breeding in the next year, given survival.
3. Stage‐specific success probability γ_*j*_: the conditional probability of successfully raising a chick to fledging in the next breeding season, given survival and breeding.

Note that while the vital rates may, in general, vary with stage *j* and time *t*, we include only the stage subscript in the following notation for clarity, where *j* corresponds to the life cycle state (*j* = 1, …, 4).

### 3.2 A Markov chain formulation of the life cycle

The life cycle of the southern fulmar (Figure 1) defines the transition structure of a finite‐state Markov chain with death as an absorbing state (Caswell, 2001, 2006). Additional absorbing states can be incorporated to calculate breeding intervals, and age at first reproduction (see next section). The transition matrix for the absorbing Markov chain is given as:

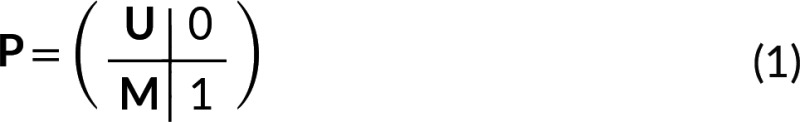

where **U** contains probabilities of transition and survival for living individuals and **M** includes the probabilities *m*_*ij*_ that an individual in a transient state *j* enters the absorbing state *i*. Based on defined breeding stages and vital rates (survival, breeding and breeding success), **U** is given by:

(2) $$U=\left(\begin{array}{cccc} \left(1-\beta_{1}\right)\sigma_{1} & 0 & 0 & 0\\ \sigma_{1}\beta_{1}\gamma_{1} & \sigma_{2}\beta_{2}\gamma_{2} & \sigma_{3}\beta_{3}\gamma_{3} & \sigma_{4}\beta_{4}\gamma_{4}\\ \sigma_{1}\beta_{1}\left(1-\gamma_{1}\right) & \sigma_{2}\beta_{2}\left(1-\gamma_{2}\right) & \sigma_{3}\beta_{3}\left(1-\gamma_{3}\right) & \sigma_{4}\beta_{4}\left(1-\gamma_{4}\right)\\ 0 & \sigma_{2}\left(1-\beta_{2}\right) & \sigma_{3}\left(1-\beta_{3}\right) & \sigma_{4}\left(1-\beta_{4}\right) \end{array}\right).$$

In Figure 1, there is only a single absorbing state: death, thus **M** is a vector of dimension 1 × *s* whose entries are the probabilities of dying for each breeding state. If there are *a* absorbing states, **M** is of dimension *a* × *s*. As in previous studies (Caswell, 2001, 2009), the transition matrix **P** is column‐stochastic (i.e. its entries are greater or equal to zero, and the sum of the entries in each column is equal to 1). It operates on column vectors, rather than the row‐stochastic matrix and row vectors common in much of the literature on Markov chains.

### 3.3 Parameter estimation: A finite mixture model for unobserved heterogeneity

To estimate vital rates σ_*j*_, β_*j*_ and γ_*j*_, we developed a multistate mark–recapture (MSMR) model (Lebreton, Nichols, Barker, Pradel, & Spendelow, 2009). Specifically, we used a finite mixture model that accounts for UH in each vital rate (Pradel, 2005; Hamel et al., 2017; Supporting Information 1). Finite mixture models allow to define a finite number *g* of groups (hidden states) in the population a priori and provide estimates of vital rates for each group separately (Supporting Information 1). In this case, each individual belongs to a given UH group for its entire life, but can change stages through life.

Mixture MSMR models also estimate the proportion of sampled individuals falling into each heterogeneity group. We denoted this distribution (the *mixing distribution*) by the *g* × 1 probability vector ***π***. Following Peron et al. (2010), we allowed for UH in all vital rates and detection probabilities. We used multimodel inference to derive a set of parameters, including UH, using model averaging as explained below (Burnham & Anderson, 2002; Lebreton et al., 2009).

#### 3.3.1 Statistical models

The structure of the MSMR model depends on the number of UH groups (Supporting Information 2). Figure S1 describes the structure of the MSMR model for *g* = 2 UH groups and *s* = 4 breeding states. In that case, there are nine possible states: *sg* = 8 alive states and one dead state, with 12 associated vital rates pertaining to each group (σ_*j*_, β_*j*_ and γ_*j*_ for breeding states *j* = 1, …, *s*).

To determine the number of UH groups and identify the vital rates that differ among groups, we used a multistep model selection approach (Burnham & Anderson, 2002) based on the Akaike information criterion (*AIC*) as described in Supporting Information 3. All analyses were conducted in the e‐surge software (Choquet, Rouan, & Pradel, 2009).

#### 3.3.2 Model selection

First, we considered a set of MSMR models including UH in each vital rate separately (Supporting Information 3.1). The ultimate number of groups was selected by applying a mixture model accounting for either one, two or three UH groups for each vital rate based on model selection. The lowest AIC values (i.e. the best performing models) retained three UH groups for vital rates of pre‐breeders and successful breeders, and two groups for vital rates of non‐breeders (Table S1). For failed breeders, the model with the lowest AIC supported three UH groups for success probability, but only two UH groups for survival and breeding probability.

From there, we considered a set of MSMR models including UH in several vital rates simultaneously (Supporting Information 3.2). All models were eventually fit using three UH groups, but for failed breeders and non‐breeders, two of the parameters were constrained to be equal (i.e. two UH groups). In the following discussion, we will refer to the three selected UH groups as UH‐1, UH‐2 and UH‐3.

The best performing models selected as measured by Δ*AIC* comprised 90% of the overall AIC weight among the set of models tested. All six models included UH in all vital rates of pre‐breeders (Table S2). Five of the six models included UH in all vital rates of successful breeders (84% of the overall AIC weight). UH in breeding probabilities of non‐breeders was included in five of the top six models (74% of the AIC weight). UH in survival probability of failed breeders was included in four out of the top six models (55% of the AIC weight). We used model averaging to generate a set of parameter estimates based on these top performing models (Burnham & Anderson, 2002).

#### 3.3.3 Results: Estimated mixing distributions and vital rates

The model‐averaged vital rates are shown in Table 1. Time‐varying parameter estimates and their associated confidence intervals are shown in Supporting Information 4. Successful breeders have a higher probability of breeding and successfully raising a chick in the following breeding season than individuals in the other breeding states. Failed breeders and non‐breeders have similar low probabilities of breeding success. Non‐breeders have the lowest adult survival and adult breeding probabilities of any of the stages. The probability of first‐time breeding by pre‐breeders is lower than the breeding probability of other stages because of delayed recruitment age.

**Table 1:** Parameter estimates obtained from model averaging of the six best performing models (i.e. Δ*AIC* < 3, total of AIC weights >90%). Estimates are for ordinary sea ice conditions as defined by Jenouvrier et al. (2015)

Vital rate	State	UH‐1	UH‐2	UH‐3
Survival	PB	1.00	0.92	1.00
Survival	S	0.93	0.99	0.89
Survival	F	0.94	0.93	0.93
Survival	NB	0.88	0.88	0.88
Breeding	PB	0.10	0.01	0.16
Breeding	S	0.96	0.80	0.97
Breeding	F	0.81	0.80	0.80
Breeding	NB	0.42	0.55	0.55
Success	PB	0.81	0.69	1.00
Success	S	0.80	0.85	0.99
Success	F	0.65	0.64	0.66
Success	NB	0.66	0.66	0.66

Beyond these general patterns, vital rates differ among UH groups, each of which can be thought of as a distinct life‐history complex. Pre‐breeders in UH‐1 and UH‐3, and successful breeders in UH‐2 have the highest survival probability across all stages and groups considered. Among pre‐breeders, individuals in UH‐3 have the highest survival, recruitment and breeding success probabilities, while those in UH‐2 have the lowest, with a very low probability of recruitment (β_1_ = 0.01). Pre‐breeders in UH‐1 have the same probability of survival as those in UH‐3, but lower probabilities of breeding and recruitment.

Among successful breeders, individuals in UH‐2 have the highest survival probability, but experience the lowest breeding probability, while individuals in UH‐3 have the lowest survival probability, but the highest breeding and success probabilities. Successful breeders in UH‐1 have the lowest breeding success but achieve a high probability of breeding.

Among failed breeders and non‐breeders, differences among UH groups are small, except for the breeding probability of non‐breeders. Among non‐breeders, individuals in UH‐1 have the lowest breeding probability.

The estimated mixing distribution of pre‐breeders at fledging is

(3) $$\pi \;=\;{\left(\begin{array}{ccc} 0.14 & 0.67 & 0.19 \end{array}\right)}^{⊤}.$$

Thus, 67% of the pre‐breeding population is estimated to belong to UH‐2, 14% to UH1 and 19% to UH3.

### 3.4 Analysis: The demographic consequences of heterogeneity

Estimated UH in vital rates affects longevity, LRO, the age at first breeding and the inter‐breeding interval. To measure these effects, we calculate the expectation and variance of each of these fitness outcomes, for each of the three UH groups.

#### 3.4.1 Longevity and stage occupancy

Let **U**_*k*_ be the transient matrix for heterogeneity group *k*. The mean and the variance of the time spent in state *i*, conditional on starting in state *j*, are given by the (*i*, *j*) entries of the fundamental matrix **N**_*k*_ and the variance matrix **V**_*k*_ respectively:

(4) $$N_{k}={\left(I-U_{k}\right)}^{-1}$$

(5) $$V_{k}=\left(2\times N_{k}^{\mathrm{diag}}-I\right)N_{k}-N_{k}\circ N_{k}\;k=1,\ldots ,g$$

where ∘ denotes the Hadamard, or element‐by‐element product, and $N_{k}^{\mathrm{diag}}$ is the matrix with the diagonal entries of **N**_*k*_ on the diagonal and zeros elsewhere (see Caswell, 2001, 2006, 2009 for calculations).

The mean and variance of longevity (the time required to reach the absorbing state of death) are calculated from the fundamental matrix. Let ${\overset{¯}{\eta }}_{k}$ be a vector containing the mean longevity (i.e. the life expectancy) of individuals in each state for heterogeneity group *k*, and let *V*(***η***_*k*_) be the vector containing the variance in longevity. Then

(6) $${\overset{¯}{\eta }}_{k}^{⊤}=1^{⊤}N_{k}$$

(7) $$V\left(\eta_{k}^{⊤}\right)=1^{⊤}N_{k}\left(2N_{k}-I\right)-{\overset{¯}{\eta }}_{k}^{⊤}\circ \;{\overset{¯}{\eta }}_{k}^{⊤}\;k=1,\ldots ,g$$

(Caswell, 2009), where **1** is a vector of 1s and the superscript ⊤ denotes the transpose.

Applying Equation (4) to the estimated matrices **U**_*k*_, we obtain the fundamental matrices for each UH group:

(8) $$N_{1}=\left(\begin{array}{cccc} 10.00 & 0 & 0 & 0\\ 9.03 & 9.30 & 7.90 & 6.66\\ 2.75 & 2.54 & 3.61 & 2.20\\ 1.69 & 1.63 & 1.92 & 3.35 \end{array}\right)$$

(9) $$N_{2}=\left(\begin{array}{cccc} 11.21 & 0 & 0 & 0\\ 1.28 & 13.08 & 11.03 & 9.92\\ 0.37 & 3.32 & 4.26 & 2.91\\ 0.54 & 5.31 & 4.93 & 5.81 \end{array}\right)$$

(10) $$N_{3}=\left(\begin{array}{cccc} 6.25 & 0 & 0 & 0\\ 8.49 & 8.49 & 7.19 & 6.45\\ 0.19 & 0.19 & 1.60 & 0.54\\ 0.43 & 0.43 & 0.81 & 2.11 \end{array}\right).$$

**Figure 2:**
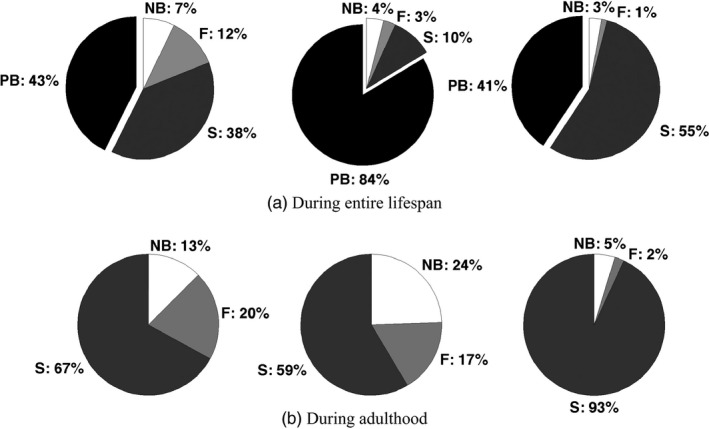
Percentages of time spent in each state during (a) the entire lifetime, and (b) the adult lifetime for individuals in each heterogeneity group from 1 (left pie chart) to 3 (right pie chart)

From the fundamental matrices **N**_*k*_, we can calculate the mean proportion of the life spent in each of the states during the entire life of the individual (Figure 2a) or during its adult life (Figure 2b). We find that individuals in each UH group experience a different life history.

1. Individuals in UH‐1 and UH‐3 spend ∼40% of their lives as pre‐breeders.
2. Individuals in UH‐2 spend most of their lives as pre‐breeders (84%).
3. Individuals in UH‐3 spend most of their lives as successful breeders (55%) than either of the other groups.

Once they reach adulthood, individuals in UH‐3 are highly successful breeders (93% of their adult lives). Adults in UH‐1 and UH‐2 differ most in the time spent non‐breeding (13% and 24% of their adult lives, respectively). They fail about 20% of their lives, compared to only 2% for the highly successful UH‐3.

**Figure 3:**
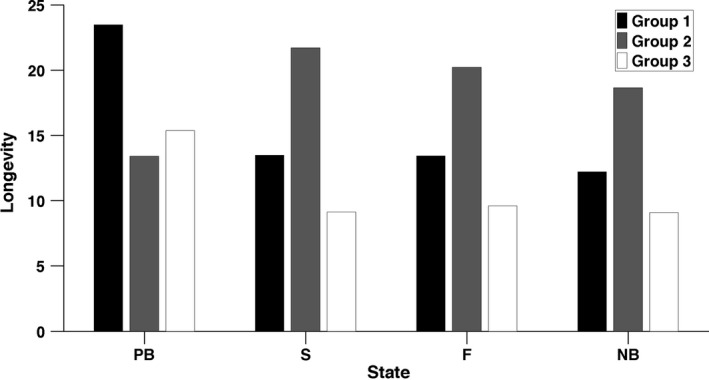
Mean longevity (i.e. life expectancy) of individuals in each stage and each unobserved heterogeneity group

The life expectancies of each state within each group are shown in Figure 3 (the variances in longevity are shown in Supporting Information 5, Table S4). At birth, individuals in UH‐1 have the longest life expectancy while individuals in UH‐2 have the shortest (Figure 3). As adults, however, we find the opposite for individuals of UH‐2, which have the longest life expectancy. These differences in life expectancy between pre‐breeder and adult states for UH‐2 reflect higher pre‐breeder mortality in group 2 (see Table 1); a hurdle that individuals that reach adulthood have already overcome. Within each UH group, life expectancy is shorter for non‐breeders than for individuals that bred (Figure 3).

#### 3.4.2 Lifetime reproductive output

The (2, 1) entry of the fundamental matrix is the expected number of successful breeding events for pre‐breeders. Because fulmars produce a single chick per breeding season, the number of successful breeding events is also the expected LRO, counting both male and female offspring (Caswell, 2009). The entry **N**(2, 1) is highlighted in bold in the fundamental matrices (8), (9), (10) and its variance is shown in Table S4.

**Figure 4:**
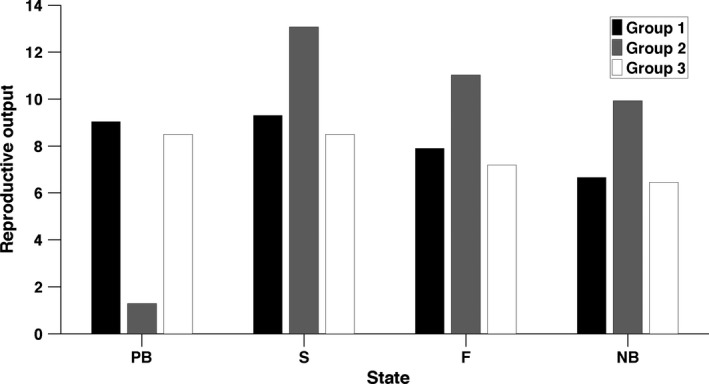
Expected lifetime reproductive output of individuals in each stage and unobserved heterogeneity group

Individuals in UH‐1 and UH‐3 produce, on average, more offspring over their lives than do individuals in group 2 (Figure 4). After reaching adulthood, however, the pattern is reversed; LRO during the adult lifetime is higher for individuals of UH‐2 than either group 1 or group 3. Within each group, expected LRO is larger for individuals that previously successfully bred and smaller for individuals that previously skipped breeding, especially among group 2 (Figure 4).

#### 3.4.3 Age at first reproduction and inter‐breeding intervals

The time required for an event to take place (e.g. breeding for the first time, breeding to one of the breeding categories) can be calculated from a life cycle model by modifying the transition matrix (1) so that the event in question becomes an absorbing state. After conditioning on eventually reaching this new absorbing state, the mean and variance of the time required to do so are calculated using the same methods used to study longevity. For a detailed description of the algorithm, see Caswell 2001, section 5.3.3).

**Figure 5:**
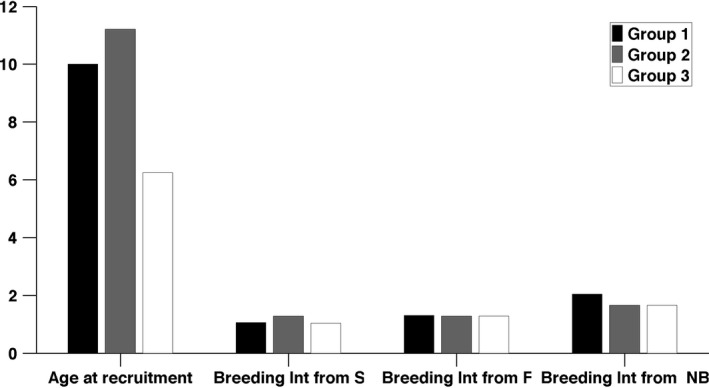
Age at first reproduction and interval to the next reproduction for individuals starting in each breeding states

We calculated the age at first reproduction as the time required for the transition from the pre‐breeder stage to either successful (stage 2) or failed (stage 3) breeding, and the inter‐breeding interval as the time required for the transition to reach either of the breeding states from each of the adult states (Figure 5, Table 2 and Table S4).

**Table 2:** Mean demographic results from the analysis of the absorbing finite‐state Markov chain for the southern fulmar for each group. Variance are shown in Table S4

Demographic results	UH‐1	UH‐2	UH‐3
Mean age 1st recruitment	10	11.2	6.2
Probability to recruit before death	1.0	0.10	1.0
Mean age 1st successful reproduction	10.3	11.7	6.25
Probability to breed successfully before death	0.97	0.10	1.00
Breeding interval:			
For previous successful breeders	1.4	1.6	1.1
For previous failed breeders	1.9	1.9	1.8
For previous non‐breeders	2.6	2.2	2.2

The mean age at first reproduction and the mean age at first successful breeding are earlier in UH‐3 than in either UH‐2 or UH‐1 (Figure 5 and Table 2). The probability of breeding successfully at least once before death is much lower for UH‐2 than other UH groups for which all individuals recruit before dying with most of them breeding successfully before dying (Table 2).

The difference among UH groups in the expected inter‐breeding interval is small (Figure 5). The interval is shorter in UH‐3 than in the other groups. Within each group, inter‐breeding intervals are slightly shorter for individuals that previously successfully bred than for individuals that previously skipped breeding.

### 3.5 The dynamics of heterogeneous cohorts

The UH groups exhibit substantial demographic differences; LRO differs by a factor of 7, age at reproduction by a factor of 1.8 and life expectancy by a factor of 1.75. These differences affect the behaviour of mixed cohorts in two ways. First, if UH affects mortality rates, as it does in our case, intra‐cohort selection will change the composition of the cohort as it ages, producing changes in apparent trajectories of survival and breeding success at the population level. Second, UH contributes to inter‐individual variance in demographic outcomes.

Here, we explore both of these effects, quantifying intra‐cohort selection and decomposing the variance in longevity, age at first reproduction and LRO into contributions from UH and individual stochasticity. Caswell (2014) and Hartemink et al. (2017), have used multistate matrix models, including UH in survival, to partition variance in longevity for human populations, but this is the first such calculation for an animal population in the wild.

The population vector for a heterogeneous cohort is a 12 × 1 vector $\tilde{n}\left(t\right)$ containing the numbers of individuals in each of the 12 combinations of stage and UH group. The vector $\tilde{n}$ is projected by the *sg* × *sg* block‐structured matrix

(11) $$\tilde{U}=K^{⊤}DKU$$

U is a block‐diagonal matrix containing the **U**_*i*_ on the diagonal and the matrix **U**_*i*_ is of dimension *s* × *s* whose entries are probabilities of transitions and survival for living individuals:

(12) $$U=\left(\begin{array}{ccc} U_{1} & \cdots & 0\\ \vdots & \ddots & \vdots \\ 0 & \cdots & U_{g} \end{array}\right)$$

and D is a block‐diagonal matrix containing the **D**_*i*_ on the diagonal and the matrix **D**_*i*_ is of dimension *g* × *g* whose entries are probabilities of transitions among heterogeneity groups:

(13) $$D=\left(\begin{array}{ccc} D_{1} & \cdots & 0\\ \vdots & \ddots & \vdots \\ 0 & \cdots & D_{s} \end{array}\right)$$

In cases like the present one, where heterogeneity is fixed, D is an identity matrix. The matrix **K** is a vec‐permutation matrix that rearranges the entries of the population vector to permit the use of the block diagonal matrices (Caswell, 2009, 2014).

**Figure 6:**
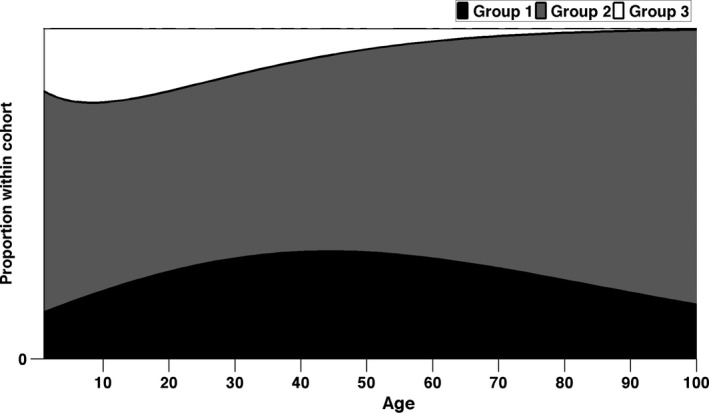
Proportion of individuals that survive to age *x* (*x*‐axis) for each group within an heterogeneous cohort

The initial cohort is composed of individuals in the pre‐breeder state, distributed among the UH groups in the proportions given by the mixing distribution **π**, from Equation (3). From this initial condition, we projected the cohort for 100 years, and show the proportional composition in Figure 6. Over the first few years, UH‐2, which has the lowest life expectancy at birth, decreases in frequency relative to UH‐1 and UH‐3. Eventually, however, this trend is reversed; UH‐3 disappears from the cohort, as does UH‐1, more slowly. Asymptotically, the cohort is composed exclusively of UH‐2. Supporting Information 6 details the dynamic of the cohort by breeding states and UH groups.

Although projection to age 100 may be unrealistic for this state‐classified model (only about 0.1% of the cohort would remain alive at this point), this is a reminder that the results of intra‐cohort selection cannot be inferred from any single demographic difference among groups. Although UH‐2 has the lowest life expectancy at birth, it eventually dominates the cohort because it has the highest adult life expectancy, and this advantage is decisive in the long run.

### 3.6 Variance decomposition: Stochasticity vs. heterogeneity

Decomposition of variance into components due to individual stochasticity and UH proceeds following (Caswell, 2009, eq. 90), based on results in probability theory (e.g. Rényi, 1970, p. 275, theorem 1), which form the basis for the analysis of variance. For any variable *ξ*, the inter‐individual variance *V*(*ξ*) can be written

(14) $$V\left(\xi \right)=E_{\pi }\left[V(\xi_{i})\right]+V_{\pi }\left[E(\xi_{i})\right]$$

where *E*_**π**_ and *V*_**π**_ denote the expectation and variance calculated over the mixing distribution **π**, and *ξ*_*i*_ is the outcome variable within group *i*. That is, the variance in *ξ* is equal to the weighted mean of the variances in each group plus the weighted variance of the group means.

The first term in Equation (14) is the within‐group variance, and is due to individual stochasticity. It captures the variance among individuals each of which experiences exactly the same stage‐specific probabilities. These variances are calculated from the Markov chain formulation of the life cycle model, as described above. The second term in Equation (14) is the between‐group variance; it is due to the differences in vital rates among the UH groups. In the absence of UH, this component is zero.

The results of applying Equation (14) to the variances in longevity, LRO, and age at first breeding are shown in Table 3. The contribution of UH to the inter‐individual variance depends on which demographic trait is considered. About 4% of the variance in age at first reproduction, 6% of the variance in longevity and 22% of the variance in LRO is due to UH. The complement (96%, 94% and 78%, respectively) is due to individual stochasticity.

**Table 3:** Variance components for longevity, LRO (lifetime reproductive output), and age at first reproduction. The within‐group component due to individual stochasticity and the between‐group component due to heterogeneity are shown, along with the percent of the variance due to heterogeneity

Variance component	Longevity	LRO	Age at first reproduction
Within‐group (stochasticity)	188.7 a^2^	43.5 a^2^	95.5 a^2^
Between‐group (heterogeneity)	11.7 a^2^	12.3 a^2^	3.6 a^2^
Percent due to heterogeneity	5.9%	22.0%	3.7%

## 4 DISCUSSION

Life‐history traits, how they combine within the lifetime of an individual to define age at first reproduction, LRO, longevity and how these traits might evolve within cohorts and across generations have been extensively studied by ecologists. The impact of UH in vital rates has further been studied by human demographers for decades (Vaupel et al., 1979; Yashin & Iachine, 1995), but has only recently attracted the attention of population ecologists (Aubry et al., 2009, 2011; Cam et al., 2002; Cam et al., 2016; Caswell, 2014; Chambert et al., 2013, 2014; Fox et al., 2006; Johnson, Burnham, & Nichols, 1986; Vindenes, Engen, & Saether, 2008; Weladji et al., 2008; Wintrebert et al., 2005). Most studies have investigated the impact of UH on a single vital rate. The simultaneous impacts of UH in both survival and reproductive traits have rarely been investigated (but see Fay, Barbraud, Delord, & Weimerskirch, 2017; Lindberg, Sedinger, & Lebreton, 2013; Plard et al., 2015 for specific vital rates and integrative demographic outcomes). We show that UH can influence life‐history traits, trade‐offs among them and inter‐individual variance in long‐lived vertebrates. Our partition of variance has shown for the first time that the contributions of individual stochasticity and UH differs among recruitment, reproduction and survival. In these cases at least, individual stochasticity contributes more to variance than does UH.

### 4.1 A diversity of life histories and trade‐offs revealed

Heterogeneity is ubiquitous in vertebrate populations due to variability in quality across individuals (Wilson & Nussey, 2010) and in their ability to acquire the resources needed to survive and reproduce (Lomnicki, 1988). In the case of the southern fulmar, UH causes substantial variability in vital rates (stage‐specific probabilities of survival, breeding and success) among three UH groups. The three UH groups define three different life‐history *complexes*. These life‐history differences would have gone undetected had we not accounted for hidden states in the first place (Jenouvrier et al., 2003, 2015), emphasizing the importance of accounting for UH, and doing so in all vital rates, not just survival.

The population contains individuals with higher (complexes 1 and 3) and lower LRO at birth (complex 2, Figure 3) but with lower (complexes 1 and 3) and higher LRO at adulthood (complex 2, Figure 4). This dichotomy between early life and adulthood is also found in longevity, with individuals in complex 2 having a shorter life expectancy at birth but longer life expectancy at adulthood than other complexes.

These three life‐history complexes are reminiscent of the gradient of life‐history strategy observed among species (i.e. the slow‐fast continuum; in birds: Saether and Bakke, 2000; in mammals: Bielby et al., 2007; Gaillard & Yoccoz, 2003; Gaillard, Festa‐Bianchet, & Yoccoz, 1998; Jones et al., 2008; Oli, 2004), which finds its roots in the classic, although somewhat obsolete, concept of *r*‐ and *K*‐selection (Dobson, 2007; Pianka, 1970):

1. Complex 1 (14% at fledging) consists of individuals with slow‐paced life histories, with a delayed but high probability of recruitment (Figure 5) and extended reproductive life span (Figure 2).
2. Complex 2 (67% at fledging) consists of individuals that are less likely to reach adulthood, recruit late and skip breeding often. They experience the highest adult survival rate across all UH groups, which is typical of a slow‐paced life history where skipped breeding is used as a strategy to conserve energy and reallocate it to adult survival rather than reproduction.
3. Complex 3 (19% at fledging) consists of individuals with fast‐paced life histories, in which individuals recruit early and attempt to breed often. They are likely to raise their offspring successfully, but experience a relatively short life span.

This diversity of life histories in the southern fulmar also reveals a diversity of life‐history trade‐offs, which are only expressed once UH differences are accounted for. Individuals in complex 3 spend most of their life as successful breeders (Figure 2). They have the highest recruitment, adult breeding probability and success probability, but the lowest adult survival, suggesting that they allocate their energy to successfully raising a chick at the expense of their own survival (i.e. trade‐offs between breeding success and future survival, Table 1). Trade‐offs between current breeding success and future survival also appear in complex 1, in which individuals are likely to attempt breeding but often fail to breed successfully, which seems positively correlated with an increased chance of survival and longevity compared to complex 3. Finally, individuals in complex 2 spend most of their life as pre‐breeders, and likely die before they have a chance to recruit. The few that survive this hurdle experience higher survival but lower breeding probability than other groups, suggesting they skip breeding to avoid jeopardizing their own survival (i.e. trading‐off between current survival and future reproduction).

### 4.2 The demography of heterogeneous cohorts

A cohort is a mixture of individuals that belong to different life‐history complexes. Within‐cohort, selection changes the composition of the cohort; initially, complexes 1 and 3 increase in frequency because they have higher juvenile survival, but eventually they are replaced by complex 2, with its higher adult longevity.

On average, the longevity of an individual that belongs to such an heterogenous cohort is ∼15 years, with an LRO of 3.7 offspring, and average recruitment at ∼10 years. However, we detected substantial variance in these demographic outcomes (Table S4), and recognize that both stochastic events and UH among individuals generate such variations in demographic outcomes (Caswell, 2011; Steiner & Tuljapurkar, 2012). Whether UH among individual results from heritability or plasticity in life‐history traits remains an open question.

Few studies have disentangled the role of UH vs. individual stochasticity in the evolution of life histories. In experimental studies, populations of genetically identical nematodes *Caenorhabditis elegans* show large variations in age at death (Sánchez‐Blanco & Kim, 2011) and lifetime reproduction (Caswell, 2011) driven by individual stochasticity. In a preliminary analysis of laboratory studies of short‐lived invertebrates, Caswell (2014) found that UH accounted for 46% to 83% of the variance in longevity. In human populations, however, UH only accounts for about 2–10% of the variance in longevity (Caswell, 2014; Hartemink et al., 2017). An finite mixture analysis of a set of laboratory life table experiments for invertebrates has found about 35% of the variance in longevity to be due to UH. For the southern fulmar, the fraction of the variance in longevity explained by UH is similar to that in human studies. It is tempting to argue that the amount of UH may relate to life expectancy, but further empirical investigation across a broader spectrum of life histories would be needed to make this claim.

Our analysis calculates the variance in LRO implied by the demographic model and its vital rates, including the estimated pattern of UH. We find that most of the variance in LRO is attributable to individual stochasticity. An additional perspective on this issue is provided in studies that also provide empirical measurements of the variance in LRO, derived from lifelong studies of identical individuals. Several previous studies, using models that did not include UH, have found that the variance predicted by individual stochasticity is sufficient to explain most or all of the observed variance in LRO in studies of seabirds (kittiwake: Steiner et al., 2010; mute swan: Tuljapurkar & Steiner, 2010; or northern fulmar: Orzack, Steiner, Tuljapurkar, & Thompson, 2010) and other species (Caswell, 2011; Tuljapurkar et al., 2009).

Steiner et al. (2010) interpreted their simulations as a neutral model for variance in LRO. The agreement of a neutral model with an empirical measurement does not show that the process is in fact neutral; it implies that the variance alone is not evidence for heterogeneity, because the variance can be explained equally well without heterogeneity. Analyses of demographic models that include heterogeneity and permit comparison with observed variances will be important. In our study, we found that 22% of the variance in LRO is attributable to fixed UH suggesting that some of the variability in life histories is not necessarily neutral.

A smaller fraction of the variance in the age at first reproduction was explained by UH (3.7%) in comparison to LRO and longevity. To our knowledge, this is the first comparison of the relative amount of variability explained by UH vs. individual stochasticity across life‐history components. Interestingly, Jenouvrier et al. (2015) found that recruitment probability is the demographic trait under the strongest selection, followed by survival probabilities while the selection gradient on the breeding success is weak. If, and it is a big if, the differences among UH groups have a genetic basis, then it may not be surprising that the variance in age at first reproduction shows little contribution from UH, because selection would have reduced the amount of genetic variation in that particular trait. Additional studies are needed to draw broader conclusions on the role UH plays in shaping life histories, and to assess whether the opposite pattern to our findings may occur in short‐lived species (i.e. larger contribution of UH to variance in longevity than variance in LRO).

### 4.3 Conclusions

Our study confirms that UH can alter not only vital rates such as survival, but also all reproductive traits, with consequences over the lifetime of individuals for recruitment age, LRO, longevity and cohort dynamics. In the southern fulmar, a rigorous statistical estimate of the amount of UH in the vital rates revealed a diversity of life‐history complexes within the population, as well as trade‐offs among life‐history traits that would have gone undetected had we not accounted for UH. The gradient of life‐history strategies observed among species should be revisited and individual differences accounted for. In addition, the respective role of UH vs. individual stochasticity varies greatly among demographic outcomes, all of which are components of fitness. Making general inferences about such patterns requires further studies across a broader range of species and ecosystems.

## 5 ACKNOWLEDGEMENTS

We thank all the field workers who participated in the long‐term study since 1964. We acknowledge Institute Paul Emile Victor (Programme IPEV 109), and Terres Australes et Antarctiques Françaises for logistical and financial support in Terre Adélie. S.J. acknowledges support from Ocean Life Institute and WHOI Unrestricted funds, and NSF projects DEB‐1257545 and OPP‐1246407. The study is a contribution to the Program EARLYLIFE funded by a European Research Council Advanced Grant under the European Community's Seven Framework Program FP7/2007‐2013 (Grant Agreement ERC‐2012‐ADG_20120314 to Henri Weimerskirch), and to the Program INDSTOCH funded by ERC Advanced Grant 322989 to Hal Caswell. We acknowledge Dominique Besson and Karine Delord for fulmar data management, Emmanuelle Cam, Tom MiIller, Jim Nichols and Guillaume Peron for constructive discussions and four anonymous reviewers for helpful comments. The Ethics Committee of IPEV and Comité de l’Environnement Polaire approved the field procedures.

## AUTHORS’ CONTRIBUTIONS

S.J. and H.C. conceived the ideas, designed methodology and obtained funding for the analyses; C.B. and H.W. collected the data and obtained funding for field work; S.J. analysed the data; S.J. led the writing of the manuscript with L.M.A. and H.C. All authors contributed critically to the drafts and gave final approval for publication.

## DATA ACCESSIBILITY

Data are archived at Dryad Digital Repository https://doi.org/10.5061/dryad.j6q05 (Jenouvrier, Aubry, Barbraud, Weimerskirch, & Caswell, 2017).
