# Supplementary material for: Interacting effects of unobserved heterogeneity and individual stochasticity in the life history of the southern fulmar
Source: J Anim Ecol. 2017 Oct 10;87(1):212–22. doi: 10.1111/1365-2656.12752 (PMC5765524; doi:10.1111/1365-2656.12752)
Supplement: Supplementary file 1 [file JANE-87-212-s001.pdf]

Supporting information: Interacting effects of unobserved heterogeneity and individual stochasticity in the life history of the Southern fulmar.

Stéphanie Jenouvrier[1a,b], Lise Aubry[c], Christophe Barbraud [b], Henri Weimerskirch[b] and Hal Caswell[d,a]

[a]Biology Dept., MS-50, Woods Hole Oceanographic Institution, Woods Hole, MA 02543, USA
[b]Centre d'Etudes Biologiques de Chizé, UMR 7372 CNRS / Univ La Rochelle- 79360 Villiers en Bois, France
[c]Wildland Resources Dept. and the Ecology Center, Utah State University, Logan, UT 84322-5230, USA)
[d]Institute for Biodiversity and Ecosystem Dynamics, University of Amsterdam, PO Box 94248, 1090 GE Amsterdam, The Netherlands

*Corresponding author: sjenouvrier@whoi.edu

[1]sjenouvrier@whoi.edu

Outline of the main study

1 Introduction

2 Study species: the Southern fulmar

3 Demography and heterogeneity

- 3.1 The fulmar life cycle
- 3.2 A Markov chain formulation of the life cycle
- 3.3 Parameter estimation: a finite mixture model for unobserved heterogeneity
  - 3.3.1 Statistical models
  - 3.3.2 Model selection
  - 3.3.3 Results: Estimated mixing distributions and vital rates
- 3.4 Analysis: the demographic consequences of heterogeneity
  - 3.4.1 Longevity and stage occupancy
  - 3.4.2 Lifetime reproductive output
  - 3.4.3 Age at first reproduction and inter-breeding intervals
- 3.5 The dynamics of heterogeneous cohorts
- 3.6 Variance decomposition: stochasticity vs. heterogeneity

4 Discussion

- 4.1 A diversity of life histories and trade-offs revealed
- 4.2 The demography of heterogeneous cohorts
- 4.3 Conclusions

Outline of the Supplementary material

1 Supplementary methods: mixture models

2 Supplementary methods: absorbing finite-state Markov chain model

3 Supplementary results: model selection

3.1 Model selection of the appropriate number of UH groups for each vital rate

3.2 Model selection accounting for UH in various vital rates simultaneously

3.3 Model selection accounting for 4 groups of UH

4 Supplementary results: parameter estimates and associated confidence intervals

5 Supplementary results: variance in life-history outcomes

6 Supplementary results: the dynamics of heterogeneous cohorts

7 Other data available on Dryad

1 Supplementary methods: mixture models

Finite mixture modeling is now a standard tool in demography since Heckman's influential work in the 1980s in econometrics (see review of Cam, Aubry & Authier (2016)). In ecology and evolution, however, these models are not as widely used (except in capture-recapture studies, e.g. Pledger, Pollock & Norris (2003); Pradel (2005); Peron *et al.* (2010)). While in medicine and other fields, mixtures are used for the analysis of life-course data (Erosheva, Matsueda & Telesca, 2014), in ecology and evolution, they are rarely applied to assess the diversity of life history trajectories occurring within an heterogeneous population (Hamel, Yoccoz & Gaillard, 2016). To account for within and between individual variations, ecologists typically use mixed models with individual random effects that are derived from continuous distributions. Results that quantify the role unobserved heterogeneity plays in shaping life histories depend on the methodological framework considered (Cam, Aubry & Authier, 2016). Here we discuss briefly why we focused on a discrete rather continuous description of unobserved heterogeneity.

There are no standard methods that can accommodate continuously distributed unobserved heterogeneity in multivariate outcomes such as the entire transition matrix that we use in our study. Models with continuously distributed heterogeneity require (1) a parametric description of the rates (e.g. the Gompertz mortality function frequently used in human demography); (2) a parametric assumption for the distribution of such heterogeneity (e.g. the gamma distribution assumed in the gamma-Gompertz frailty model); and (3) an assumption of how heterogeneity affects these rates (e.g. the proportional hazard assumption used by Vaupel, Manton & Stallard (1979)). If the vital rate model is changed (e.g. the Gompertz-Makeham model instead of the Gompertz model), or if the heterogeneity distribution is changed (e.g. a log-normal distribution instead of a gamma distribution), or if the heterogeneity effects are changed (e.g. a variable rate effect instead of a proportional hazard), the conclusions might end up being completely different.

The great advantage of finite mixture models is that unobserved heterogeneity groups can be fit to the data, compared using model selection, without any a priori assump-

tions regarding how many groups might be present in the population. These models then provide estimates of how such heterogeneity affects all of the vital rates considered. In mark-recapture studies, statisticians have developed and even compared the performance of survival models that accomodate either continuously distributed heterogeneity, or heterogeneity treated as finite mixtures. Pledger & Schwarz (2002) ultimately concluded that two mixtures models (i.e. two groups of UH in the population) often provides a better representation of UH in survival than continuously distributed heterogeneity models would.

Recently, Hamel, Yoccoz & Gaillard (2016) performed simulations in an effort to compare the precision and accuracy of estimates obtained from mixture models versus mixed models under a wide range of ecological contexts. They found that mixture models performed well in quantifying the underlying unobserved heterogeneity in vital rates within an heterogeneous population in most cases, and that Akaike information criterion (AIC) selected the right number of UH groups in most ecological situations (see also Cubaynes *et al.* (2012)).

2 Supplementary methods: absorbing finite-state Markov chain model

To parameterize the absorbing finite-state Markov chain model, we used multi-event mixture models that account for unobserved heterogeneity (UH) in each vital rate (Pradel, 2005; Hamel, Yoccoz & Gaillard, 2016). Finite mixture models allow to define a finite number g of groups (hidden states) in the population a priori and provide estimates of vital rates for each group. In our case, each individual belongs to one UH group but can change stages through life. These models distinguish between a vector of probabilities of initial presence in the various states π, a matrix $\mathbf{P}$ that describes transitions among states, and a matrix of event probabilities $\mathbf{E}$ that links the events to the states (Pradel, 2005; Choquet, Rouan & Pradel, 2009; Peron *et al.*, 2010)

Supplementary figure 1 shows the diagram describing the hidden Markov chain structure for the matrix $\mathbf{P}$ that is decomposed in three matrices:

- a matrix $\mathbf{\Sigma}$ that describes the mortality process used to estimate survival probabilities σ_k, k referring here to the combination of breeding states $j \in [1 \cdots s = 4]$ and groups of UH $i \in [1 \cdots g]$, with $k \in [1 \cdots s \times g]$;
- a matrix $\mathbf{\Psi}$ that describes the breeding process – returning to the breeding site and laying an egg– to estimate breeding probability β_k;
- a matrix $\mathbf{\Gamma}$ that describes the rearing process – successfully raising a chick – to estimate breeding success probability γ_k.

The observer records or "events" at season t are:

- not seen,
- seen as pre-breeder, i.e. has not yet been observed brewing at t: fledged chicks in season t and older individuals at the colony that have not yet bred;
- seen as successful breeder at t: successfully raised a chick in season t,

- seen as failed breeder at t: failed to hatch an egg or raise a chick in season t,
- seen as non-breeder, i.e. has not been observed breeding in season t: individuals at the colony that have not been seen laying, incubating an egg, or raising a chick.

The following files available on Dryad describe the matrix formulation used in software E-surge (Choquet, Rouan & Pradel, 2009) (note that they are row-stochastic matrices):

- `Hetero2classes.pat`: describes the matrix formulation with 2 UH groups (Fig. 1) used in GEPAT of software E-surge.
- `Hetero3classes.pat`: describes the matrix formulation with 3 UH groups.
- `Hetero4classes.pat`: describes the matrix formulation with 4 UH groups.

Supplementary figure 1 describes the matrix formulation with 2 UH groups.

1. At the beginning of the annual cycle, individuals are classified into $sg+1 = 9$ states; supplementary figure 1 illustrates whether individuals within each state survived the entire year (σ).
2. At the beginning of the breeding season, survivors in all $sg = 8$ states are present in the population. Individuals may return to the colony to breed (β). $2g = 4$ additional states are added (10 to 13) to track individuals which have previously bred and did not breed in the focal year (12 and 13), as well as pre-breeders which did not breed that year (10 and 11).
3. During the breeding season, these $sg + 1 + 2g = 13$ states are present in the population and individuals present and breeding at the colony (states 1 to 8) may successfully raise a chick through fledging (γ).
4. At the end of the breeding season, individuals who skipped breeding (10 to 13 states) are reassigned to either the pre-breeder or non-breeder state.

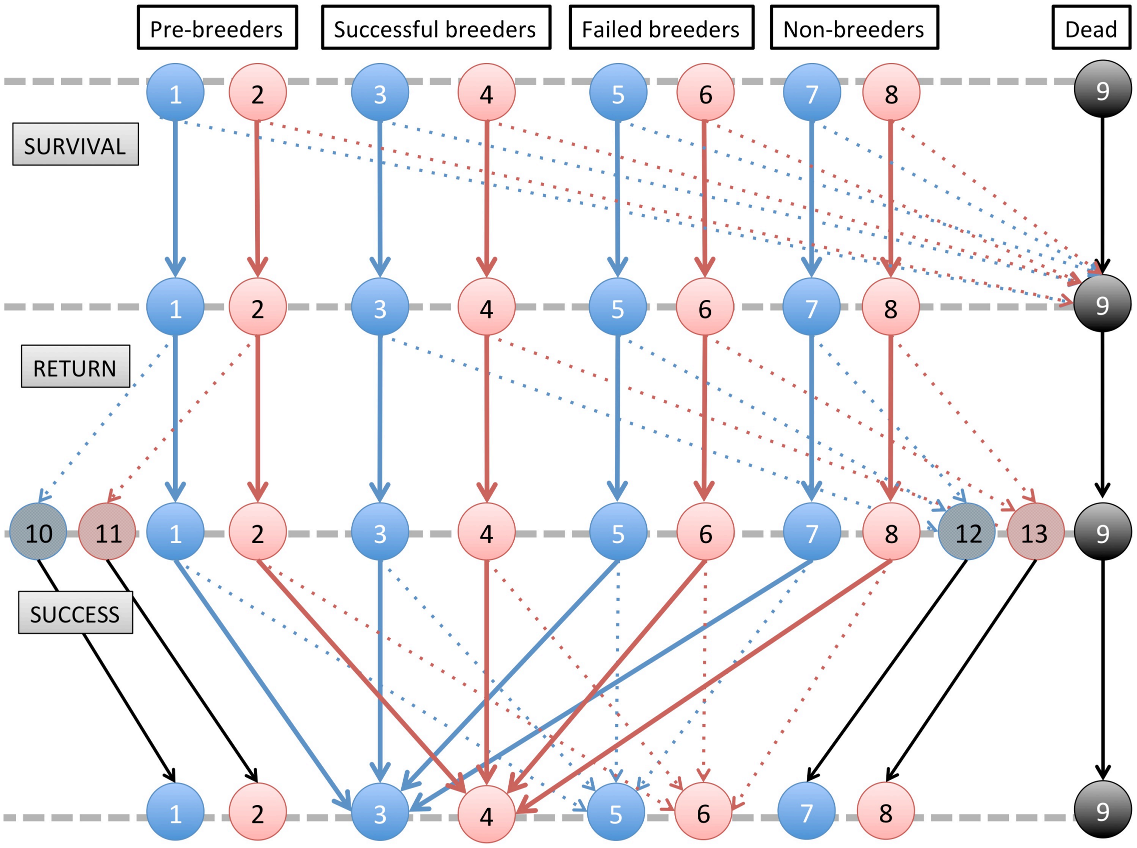

Figure 1: Diagram describing the hidden Markov chain structure for a model with two UH groups. Circles represent the possibles states (a combination of breeding states s and UH groups g), with the dead state in black and the UH groups represented in blue and red. Dashed gray lines separate the different transitions: (1) the mortality process used to estimate survival probabilities σ_k, (2) returning to the breeding site and laying an egg to estimate breeding probability β_k, (3) successfully raising a chick to estimate breeding success probability γ_k (gray boxes). A black line represents a probability of 1. Thick lines are the θ_k probability (i.e. σ_k for survival, β_k for breeding and γ_k success probabilities), while dotted lines are $1 - \theta_k$. Shaded states from 10 to 13 during the second phase represent individuals that do not return to the breeding site – 10 and 11 are pre-breeders while 12 and 13 are adults. The matrix description of this model is provided on Dryad in the pattern file of the software Esurge (Hetero2classes.pat).

3 Supplementary results: model selection

Jenouvrier et al. (2015) previously estimated vital rates of the Southern fulmar without accounting for unobserved heterogeneity in vital rates (UH); we used the same dataset in this analysis (n = 1165 known age individuals from 1964 to 2010). We performed model selection by testing a candidate set of models and comparing them using Akaike's information criterion (AIC). Model(s) with the lowest AIC that accounted for 90% of AIC weights were selected and used to eventually compute model averaged estimates (Burnham & Anderson, 2002).

We initiated the model selection process with an umbrella model identified in Jenouvrier et al. (2015) that accounts for the following structure:

- detection probabilities among states vary additively with time[1] (p_{s+t}).
- breeding probabilities among states vary additively with time (β_{s+t}).
- success probabilities vary additively with time among states, with equal breeding success between pre-breeders, failed breeders and non-breeders $\gamma_1 = \gamma_3 = \gamma_4$ – time variations are a function of a covariate describing unfavorable and favorable extreme sea ice conditions 'SICs' across years ($\gamma_{1=3=4,2+SIC}$);
- annual adult survival probabilities do not vary with time, nor do they vary among states (σ).

A model that followed the structure of the umbrella model defined above, and that accounted for unobserved heterogeneity in all vital rates:

$p_{s+t/UH}, \beta_{s+t/UH}, \gamma_{1=3=4,2+SIC/UH}, \sigma_{UH}$

was overparameterized and did not converge.

Given the problem of model convergence and identifiability of parameters when estimating multiple vital rates with hidden states, we followed a pragmatic approach that divided the model selection process into 3 steps:

[1] Additive time-variation requires parameters to vary in parallel on the logit scale.

1. we analyzed a set of models that account for 1, 2, or 3 groups of UH for each vital rate to select the appropriate number of UH groups in a given vital rate, while the non focal parameters followed the structure of our umbrella model $p_{s+t}, \beta_{s+t}, \gamma_{1=3=4,2+SIC}, \sigma$ (section 3.1).

2. we analyzed a set of models that account for UH in several vital rates simultaneously (section 3.2).

3. to check 'a posteriori' if our model selection approach of the number of UH groups was robust, we selected the best performing models from step 2 and compared them to models with a similar structure but that accounted for 4 UH groups (section 3.1).

3.1 Model selection of the appropriate number of UH groups for each vital rate

The first step in the model selection process was to assess whether variability in fulmar vital rates was best represented by 1, 2, or 3 groups of UH for each vital rate separately. Supplementary Table 1 indicated that models including 2 or 3 groups were both well supported by the data, and their associated AIC was lower than the AIC of a model without UH groups ($\triangle AIC < 0$). Models with 3 groups were selected for the vital rates of pre-breeders and successful breeders, while only 2 groups were most often best supported for the vital rates of other states. All models selected included differences in detection probabilities among groups, except for the breeding probabilities of failed breeders.

3.2 Model selection accounting for UH in various vital rates simultaneously.

The second step of the model selection process aimed at incorporating differences among UH groups for all vital rates simultaneously by bringing the selected structure together once the optimal number of UH groups in each vital rate had been identified (step 1). The first step identified 3 UH groups in each vital rate for most vital rates, and thus our selected model structure included 3 UH groups. We selected the model

Table 1: Step 1 of the model selection process: selection of the number of UH groups for each vital rate. The first column indicates the vital rate studied, and the second column represents the model selected for this particular vital rate. Subsequent columns specify the number of parameters (K), the deviance and AIC of the selected UH model. The model with no UH group has an AIC equal to 7416.366 and ΔAIC_i is the difference between this reference model and model i. A more detailed Table is provided on Dryad.

name model	UH model	K	Deviance	AIC	ΔAIC
survival PB	3 groups; detection + survival	103	7006.85	7212.85	-203.517
survival S	3 groups; detection + survival	104	7186.98	7394.98	-21.385
survival F	2 groups; detection	99	7215.31	7413.30	-3.061
survival NB	2 groups; detection + survival	100	7214.60	7414.60	-1.765
breeding PB	3 groups; detection + breeding	102	7058.91	7262.91	-153.456
breeding S	3 groups; detection + breeding	103	7178.44	7384.44	-31.926
breeding F	2 groups; breeding	98	7220.91	7416.91	0.542
breeding NB	2 groups; detection+ breeding	99	7201.66	7399.66	-16.706
success PB	3 groups; detection + success	106	7130.66	7342.66	-73.708
success S	3 groups; detection + success	103	7181.61	7387.61	-28.753
success F	3 groups; detection + success	107	7200.61	7414.61	-1.753
success NB	2 groups; detection + success	100	7217.37	7417.37	1.003

with lowest AIC (three UH groups in pre-breeder survival) and subsequently included differences among group in each vital rates (Supplementary Tables 1 and 2). If the model including the additional vital rate had a lower AIC than the previously selected model, differences between the groups in the focal vital rate were included. If not, the model selection procedure moved on to the next vital rate by constraining the focal vital rate to be equal among the 3 groups. If only 2 groups of UH were previously selected for the focal vital rate, we constrained the focal vital rate to be equal between 2 of the 3 groups. For example, individuals do not transition among the 3 groups but pre-breeders of group UH-1 have the same annual survival σ_1 than pre-breeders of group UH-3. The top 6 models selected as measured by ΔAIC comprised 90% of the overall AIC weight for the set of models tested (Supplementary Table 2).

3.3 Model selection accounting for 4 groups of UH.

The last step of our model selection showed that 4 UH groups equated to higher AIC and estimates equivalent to that of models that accounted of 3 UH groups, with $\pi_4 \simeq 0$. Specifically, supplementary Table 3 details the model selection process when 4 UH groups were included, and compares AIC between 3 and 4 UH groups for the 6 best

Table 2: Step 2 of the model selection process: selection of UH groups in all vital rates, simultaneously. The first column describes the structure of the model, i.e. the vital rates simultaneously included for 3 groups of UH. Subsequent columns indicate the number of parameters (K) and the deviance (dev) for each model considered from which we calculate AIC. △ is the difference between the model with the lowest AIC (first line) and model considered and W is the AIC weight. The AIC weight of the selected models for our model averaging estimates are shown (i.e. the top 6 models comprising 90% of model weights).

models	K	dev	$\triangle AIC$	W
σ PB + β PB+ γ PB+ β S+ γ S+σ S+ β NB	120	6913.21	0	**0.29**
σ PB + β PB+ γ PB+ β S+ γ S+σ S+ β NB +σ F	122	6909.63	0.42	**0.23**
σ PB + β PB+ γ PB+ β S+ γ S+σ S+ β NB +σ F +β F	123	6908.52	1.31	**0.15**
σ PB + β PB+ γ PB+ β S+ γ S+σ S+ β NB +σ F + σ NB	123	6909.55	2.34	**0.09**
σ PB + β PB+ γ PB+ β S+ γ S+σ S+ β NB +σ F + γ F	130	6895.88	2.67	**0.08**
σ PB + β PB+ γ PB+ β S+ γ S	115	6926.17	2.96	0.07
σ PB + β PB+ γ PB+ β S+ γ S+σ S	118	6920.96	3.75	0.04
σ PB + β PB+ γ PB+ β S+ γ S+σ S+ β NB +σ F +β F+ γ NB	125	6907.739	4.529	0.03
σ PB + β PB+ γ PB+ β S+ γ S+σ S+ β NB +σ F +γ NB	124	6910.42	5.21	0.02
σ PB + β PB+ γ PB+ β S	113	6948.81	21.6	0.00
σ PB + β PB	105	7002.24	59.03	0.00
σ PB	103	7006.849	59.639	0.00
σ PB + β PB+ γ PB	108	6997.18	59.97	0.00
σ PB + β PB+ γ PB+ β S+ γ S+σ S+ β NB +σ NB	122	6917.03	60.97	0.00

models supported by the data (Supplementary Table 2). Files available on Dryad in folder `cmrfile/6bestmodels_4groups` provide associated estimates and show that $\pi_4 \sim 0$ for all models.

Table 3: Model selection including 4 UH groups. The first and second columns describe the structure of the model and number of groups. Subsequent columns indicate the number of parameters (K), the deviance and AIC for each model considered.

age at first reproduction

models	# groups	K	dev	AIC
σ PB + β PB+ γ PB+ β S+ γ S+σ S+ β NB	3 groups	120	6913.21	7153.21
	4 groups	129	6903.69	7161.69
σ PB + β PB+ γ PB+ β S+ γ S+σ S+ β NB +σ F	3 groups	122	6909.63	7153.63
	4 groups	131	6904.43	7166.43
σ PB + β PB+ γ PB+ β S+ γ S+σ S+ β NB +σ F +β F	3 groups	123	6908.52	7154.52
	4 groups	132	6917.45	7181.45
σ PB + β PB+ γ PB+ β S+ γ S+σ S+ β NB +σ F + σ NB	3 groups	123	6909.55	7155.55
	4 groups	132	6934.81	7198.81
σ PB + β PB+ γ PB+ β S+ γ S+σ S+ β NB +σ F + γ F	3 groups	130	6895.88	7155.88
	4 groups	142	6903.58	7187.58
σ PB + β PB+ γ PB+ β S+ γ S	3 groups	115	6926.17	7156.17
	4 groups	121	6957.95	7199.95

4 Supplementary results: parameter estimates and associated confidence intervals

The estimates and their confidence intervals are available on Dryad as: `Estimate_hessian.xlsx`. To investigate parameter redundancy and identifiability, we used the model implemented in Esurge (Giménez *et al.*, 2004; Choquet, Rouan & Pradel, 2009). All our parameters are identifiable, but some are at a boundary (zero or one).

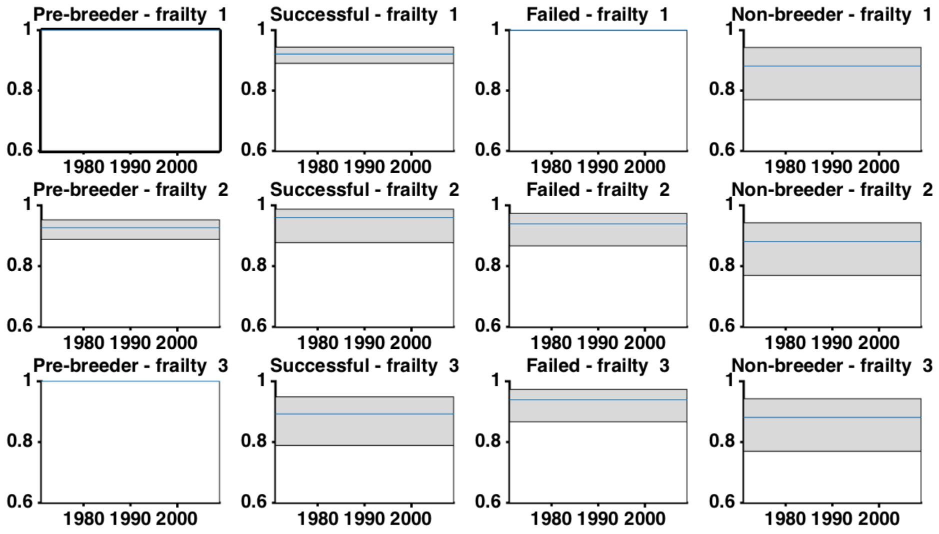

Figure 2: Adult survival probabilities (blue lines) and associated confidence intervals (gray shaded area). Estimates from the model σ PB + β PB+ γ PB+ β S+ γ S+σ S+ β NB +σ F . Estimates of pre-breeder survival for UH groups 1 and 3, as well as failed breeder survival for UH group 1 are at the boundary.

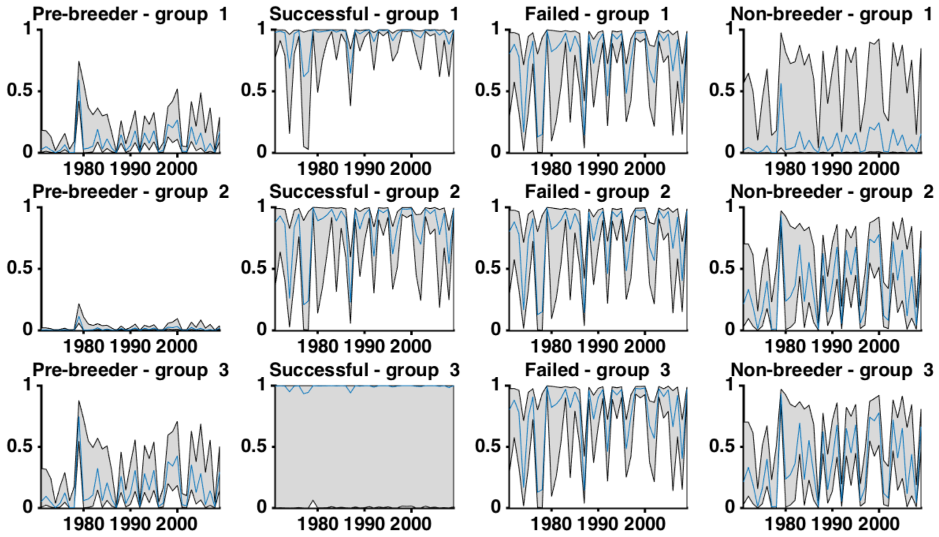

Figure 3: Breeding probabilities and associated confidence intervals.

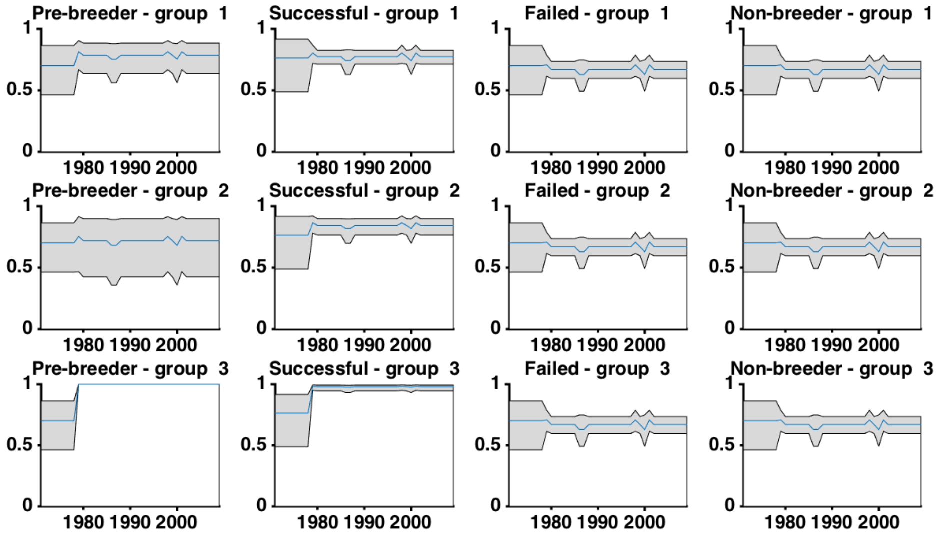

Figure 4: Breeding success probabilities and associated confidence intervals. Estimates of pre-breeder survival of UH group 3 are at the boundary.

5 Supplementary results: variance in life-history outcomes

Table 4: Mean and variance of life-history outcomes for each group of unobserved heterogeneity.

life-history outcome	Mean			Variance		
	Group 1	Group 2	Group 3	Group 1	Group 2	Group 3
Longevity at birth	23.5	13.4	15.4	253.7	198.2	107.1
Longevity at adulthood:						
for successful breeders	13.5	21.7	9.1	163.8	407.0	74.2
for failed breeders	13.4	20.2	9.6	163.1	405.8	74.9
for non- breeders	12.2	18.6	9.1	159.2	398.9	74.5
Lifetime reproductive output	9.0	1.3	8.5	77.4	30.6	63.6
Reproductive output at adulthood:						
for successful breeders	9.3	13.1	8.5	77.2	158.0	63.6
for failed breeders	7.9	11.0	7.2	76.7	155.9	63.2
for non- breeders	6.7	9.9	6.5	72.9	151.2	61.5
Mean age 1st reproduction	10.0	11.2	6.3	90.0	114.5	32.8
Birth interval:						
for previous successful breeders	1.1	1.3	1.0	0.2	0.6	0.1
for previous failed breeders	1.3	1.3	1.3	0.9	0.6	0.6
for previous non- breeders	2.0	1.7	1.7	2.1	1.1	1.1

6 Supplementary results: the dynamics of heterogeneous cohorts

The initial cohort is composed of individuals in the pre-breeder state, distributed among the UH groups in the proportions given by the mixing distribution $\boldsymbol{\pi}$. From this initial condition, we projected the cohort for 100 years, and show the proportional composition by breeding states and UH groups in Figure 5. Over the first few years, UH-2, which has the lowest life expectancy at birth and later age at recruitment, decreases in frequency relative to UH-1 and UH-3 among successful, failed and non breeders. Interestingly, within the cohort, UH-3 are the most abundant group within the successful breeder state for younger age, and UH-1 are the most abundant group within the failed breeder state for younger age. Eventually, however, this trend is reversed; UH-3 disappears from the cohort, as does UH-1, more slowly. Asymptotically, the cohort is composed exclusively of UH-2.

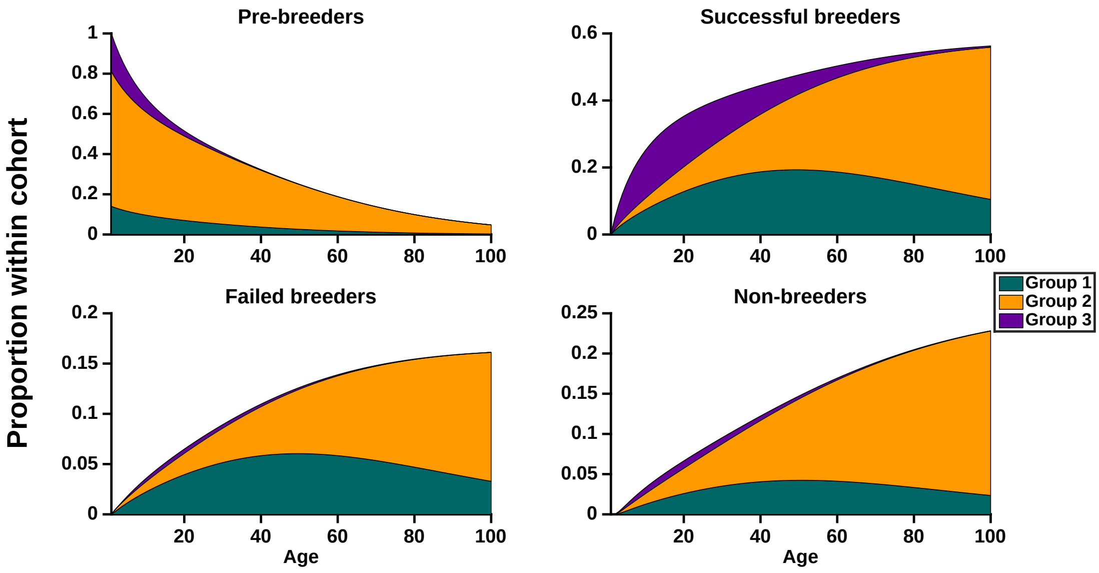

Figure 5: Proportion of individuals that survive to age x (x-axis) for each group and breeding states (panels) within an heterogeneous cohort.

7 Other data available on Dryad

`ResultMixtmodelselection1.xls`: is a detailed version of Table 1 and describes the first step of the model selection process, studying each vital rate separately.

The folder `cmrfiles` contains the detailed output of Esurge of the six best performing models, and the file `Estimate_modeaver_6best.xlsx` summarizes the estimate of these six models.

References

Burnham, K. & Anderson, D. (2002) *Model Selection and Multimodel Inference: A Practical Information-theoretic Approach.* Springer.

Cam, E., Aubry, L.M. & Authier, M. (2016) The Conundrum of Heterogeneities in Life History Studies. *Trends in Ecology & Evolution.*

Choquet, R., Rouan, L. & Pradel, R. (2009) Program E-SURGE: a software application for fitting multievent models. *Environmental and ecological statistics*, **3**, 207–215.

Cubaynes, S., Lavergne, C., Marboutin, E. & Gimenez, O. (2012) Assessing individual heterogeneity using model selection criteria: how many mixture components in capture-recapture models? *Methods in Ecology and Evolution*, **3**, 564–573.

Erosheva, E.A., Matsueda, R.L. & Telesca, D. (2014) Breaking bad: two decades of life-course data analysis in criminology, developmental psychology, and beyond. *Annual Review of Statistics and Its Application*, **1**, 301–332.

Giménez, O., Viallefont, A., Catchpole, E. & Choquet, R. (2004) Methods for investigating parameter redundancy. *Animal Biodiversity and Conservation*, **27**, 1–12.

Hamel, S., Yoccoz, N.G. & Gaillard, J.M. (2016) Assessing variation in life-history tactics within a population using mixture regression models: a practical guide for evolutionary ecologists. *Biological Reviews*, pp. 1464–7931.

Peron, G., Crochet, P.A., Choquet, R., Pradel, R., Lebreton, J.D. & Gimenez, O. (2010) Capture-recapture models with heterogeneity to study survival senescence in the wild. *Oikos*, **119**, 524–532. ISSN 0030-1299.

Pledger, S., Pollock, K.H. & Norris, J.L. (2003) Open capture-recapture models with heterogeneity: I. Cormack-Jolly-Seber model. *Biometrics*, **59**, 786–794.

Pledger, S. & Schwarz, C.J. (2002) Modelling heterogeneity of survival in band-recovery data using mixtures. *Journal of Applied Statistics*, **29**, 315–327.

Pradel, R. (2005) Multievent: An extension of multistate capture-recapture models to uncertain states. *Biometrics*, **61**, 442–447.

Vaupel, J., Manton, K. & Stallard, E. (1979) Impact of heterogeneity in individual frailty on the dynamics of mortality. *Demography*, **16**, 439–454. ISSN 0070-3370.
